# Editorial: Carbon-Based Bifunctional Oxygen Electrocatalysts

**DOI:** 10.3389/fchem.2020.00713

**Published:** 2020-09-23

**Authors:** Zexing Wu, Xiankai Wan, Wei Jin, Gengtao Fu

**Affiliations:** ^1^State Key Laboratory Base of Eco-Chemical Engineering, College of Chemistry and Molecular Engineering Qingdao University of Science and Technology, Qingdao, China; ^2^Department of Chemistry, Research Center for Materials Science, Graduate School of Science, Nagoya University, Nagoya, Japan; ^3^School of Chemical and Material Engineering, Jiangnan University, Wuxi, China; ^4^School of Chemical and Biomedical Engineering, Nanyang Technological University, Singapore, Singapore

**Keywords:** carbon, metal-air batteries, oxygen reduction reaction, oxygen evolution reaction, bifunctional oxygen electrocatalysts

With the ever-increasing environmental pollution and energy crisis, the demand for sustainable and environmentally friendly energy conversion and storage technologies has been on the rise. Recently, metal-air batteries have been extensively investigated because of their high theoretical energy density, low cost, environmental benignity, and satisfactory safety and are, thus, considered as promising energy devices (Wang et al., 2014; Fu et al., 2018). However, the sluggish kinetics of oxygen-related reactions, including the oxygen reduction reaction (ORR) and the oxygen evolution reaction (OER), hinders the large-scale practical applications of metal-air batteries (Pan et al., 2018; Wang et al., 2018) Therefore, it is necessary to exploit efficient electrocatalysts that exhibit high ORR and OER activities.

Until now, Pt- and Ir/Ru-based materials exhibit the best electrocatalytic performances for the ORR and OER, respectively. Nevertheless, their scarcity and high cost limit their large-scale employment in metal-air batteries. Therefore, it is important to exploit low-cost and earth-abundant electrocatalysts as alternatives to noble metals for the ORR and OER. Among the studied non-precious electrocatalysts, carbon-based nanomaterials have been widely investigated for oxygen electrode reactions because of their numerous advantages such as wide availability, low cost, remarkable electroconductivity, and environmental benignity. However, the electrocatalytic activities of carbon-based nanomaterials are lower than those of noble metals. Recently, various strategies have been adopted to improve the electrocatalytic performance, such as heteroatom (e.g., N, P, S, B, and F) doping and nanostructure design (including the porous structure and multi-dimensional morphology). Heteroatom doping facilitates electronic re-distribution and electroconductivity improvement, which, in turn, enhances the electrocatalytic performance (Chen et al., 2019; Zheng et al., 2019). The presence of a porous structure with multi-dimensional morphology is beneficial for promoting catalytic activity by accelerating the diffusion of the electrolyte and the release of the generated gas (Zhang et al., 2017; Hu et al., 2019). Furthermore, in addition to their application as an electrocatalyst for oxygen-related reactions, carbon-based nanomaterials are considered as ideal matrices to support metal compounds, which can prevent the aggregation of metal nanoparticles and reduce the electrical resistance. Herein, we collected six valuable contributions focusing on the preparation, structure control, and application of various carbon-based electrocatalysts. The outstanding ORR performance of nitrogen-doped porous carbon, designed and developed by (Liu et al., 2019) via the direct pyrolysis of natural cattail fibers, is discussed. The preparation methods proposed by (Wang M. et al., 2020) for various biomass-derived catalysts and their catalytic performance for the ORR in alkaline, neutral, and acidic media are summarized. Furthermore, the carbon-based materials serving as matrices to support Fe-Ni_2_P, (Xiao et al., 2019) Co_3_O_4_, (Cheng et al., 2019) Fe_1−x_S, (Wang H. et al., 2019), and Fe/N (Rauf et al., 2020) are also discussed in this Research Topic; all the prepared electrocatalysts were found to exhibit excellent electrocatalytic activities for oxygen electrode reactions. As guest editors, we would like to thank all the authors for their valuable contributions to this Research Topic and all the reviewers for their important and thoughtful comments and insights. We hope that this Research Topic will provide useful insights for the development of carbon-based bifunctional catalysts and furnish a background for the research related to advanced carbon chemistry in redox reactions.

## Author Contributions

All authors listed have made a substantial, direct and intellectual contribution to the work, and approved it for publication.

## Conflict of Interest

The authors declare that the research was conducted in the absence of any commercial or financial relationships that could be construed as a potential conflict of interest.

## References

[B1] Chen J. Fan C. Hu X. Wang C. Huang Z. Fu G.. (2019). Hierarchically porous Co/CoxMy (M = P, N) as an efficient Mott–Schottky electrocatalyst for oxygen evolution in rechargeable Zn-Air batteries. Small 15:1901518. 10.1002/smll.201901518 31140732

[B2] Cheng G. Liu G. Liu P. Chen L. Han S. Han J.. (2019). Nitrogen-doped ketjenblack carbon supported Co_3_O_4_ nanoparticles as a synergistic electrocatalyst for oxygen reduction reaction. Front. Chem. 7:766. 10.3389/fchem.2019.00766 31867304 PMC6904301

[B3] Fu G. Tang Y. Lee J.-M. (2018). Recent advances in carbon-based bifunctional oxygen electrocatalysts for Zn-air batteries. ChemElectroChem 5, 1424–1434. 10.1002/celc.201800373

[B4] Hu X. Chen Y. Zhang M. Fu G. Sun D. Lee J.-M. (2019). Alveolate porous carbon aerogels supported Co9S8 derived from a novel hybrid hydrogel for bifunctional oxygen electrocatalysis. Carbon 144, 557–566. 10.1016/j.carbon.2018.12.099

[B5] Liu Y. Hu M. Xu W. Wu X. Jiang J. (2019). Catalytically active carbon from cattail fibers for electrochemical reduction reaction. Front. Chem. 7:786. 10.3389/fchem.2019.00786 31799241 PMC6878766

[B6] Pan J. Xu Y. Yang H. Dong Z. Liu H. Xia B. (2018). Advanced architectures and relatives of air electrodes in Zn–Air batteries. Adv. Sci. 5:1700691. 10.1002/advs.201700691 29721418 PMC5908379

[B7] Rauf M. Wang J. Iqbal W. Abbas M. Khan S. A. Khan Q. U.. (2020). Novel heteroatom-doped Fe/N/C electrocatalysts with superior activities for oxygen reduction reaction in both acid and alkaline solutions. Front. Chem. 8:78. 10.3389/fchem.2020.00078 32133340 PMC7040484

[B8] Wang H. Qiu X. Wang W. Jiang L. Liu H. (2019). Iron sulfide nanoparticles embedded into nitrogen and sulfur co-doped carbon sphere as a highly active oxygen reduction electrocatalyst. Front. Chem. 7:855. 10.3389/fchem.2019.00855 31921777 PMC6920104

[B9] Wang H.-F. Tang C. Zhang Q. (2018). A review of precious-metal-free bifunctional oxygen electrocatalysts: rational design and applications in Zn–Air batteries. Adv. Funct. Mater. 28:1803329 10.1002/adfm.201803329

[B10] Wang M. Wang S. Yang H. Ku W. Yang S. Liu Z.. (2020). Carbon-based electrocatalysts derived from biomass for oxygen reduction reaction: a minireview. Front. Chem. 8:116. 10.3389/fchem.2020.00116 32185161 PMC7059099

[B11] Wang Z.-L. Xu D. Xu J.-J. Zhang X.-B. (2014). Oxygen electrocatalysts in metal-air batteries: from aqueous to nonaqueous electrolytes. Chem. Soc. Rev. 43, 7746–7786. 10.1039/C3CS60248F 24056780

[B12] Xiao Y. Deng S. Li M. Zhou Q. Xu L. Zhang H.. (2019). Immobilization of Fe-doped Ni_2_P particles within biomass agarose-derived porous N, P-carbon nanosheets for efficient bifunctional oxygen electrocatalysis. Front. Chem. 7:523. 10.3389/fchem.2019.00523 31448255 PMC6691339

[B13] Zhang W. Xu X. Zhang C. Yu Z. Zhou Y. Tang Y. (2017). 3D space-confined pyrolysis of double-network aerogels containing In–Fe cyanogel and polyaniline: a new approach to hierarchically porous carbon with exclusive Fe–Nx active sites for oxygen reduction catalysis. Small Methods 1:1700167 10.1002/smtd.201700167

[B14] Zheng X. Wu J. Cao X. Abbott J. Jin C. Wang H. (2019). N-, P-, and S-doped graphene-like carbon catalysts derived from onium salts with enhanced oxygen chemisorption for Zn-air battery cathodes. Appl. Catal. B. Environ. 241, 442–451. 10.1016/j.apcatb.2018.09.054

